# Experimental demonstration of the microscopic origin of circular dichroism in two-dimensional metamaterials

**DOI:** 10.1038/ncomms12045

**Published:** 2016-06-22

**Authors:** A. B. Khanikaev, N. Arju, Z. Fan, D. Purtseladze, F. Lu, J. Lee, P. Sarriugarte, M. Schnell, R. Hillenbrand, M. A. Belkin, G. Shvets

**Affiliations:** 1 Department of Physics, Queens College and Graduate Center of The City University of New York, Queens, New York 11367, USA; 2 Department of Physics, The University of Texas at Austin, Austin, Texas 78712, USA; 3 Department of Electrical and Computer Engineering, The University of Texas at Austin, Austin, Texas 78758, USA; 4 CIC nanoGUNE Consolider, Tolosa Hiribidea, 76, Donostia E-20018, Spain

## Abstract

Optical activity and circular dichroism are fascinating physical phenomena originating from the interaction of light with chiral molecules or other nano objects lacking mirror symmetries in three-dimensional (3D) space. While chiral optical properties are weak in most of naturally occurring materials, they can be engineered and significantly enhanced in synthetic optical media known as chiral metamaterials, where the spatial symmetry of their building blocks is broken on a nanoscale. Although originally discovered in 3D structures, circular dichroism can also emerge in a two-dimensional (2D) metasurface. The origin of the resulting circular dichroism is rather subtle, and is related to non-radiative (Ohmic) dissipation of the constituent metamolecules. Because such dissipation occurs on a nanoscale, this effect has never been experimentally probed and visualized. Using a suite of recently developed nanoscale-measurement tools, we establish that the circular dichroism in a nanostructured metasurface occurs due to handedness-dependent Ohmic heating.

## Abstract (web summary)

The effect of Ohmic dissipation in two-dimensional chiral materials has never been experimentally verified. Here, Khanikaev *et al*. demonstrate that the circular dichroism in a nanostructured metasurface occurs due to handedness-dependent Ohmic heating.

## Graphical abstract

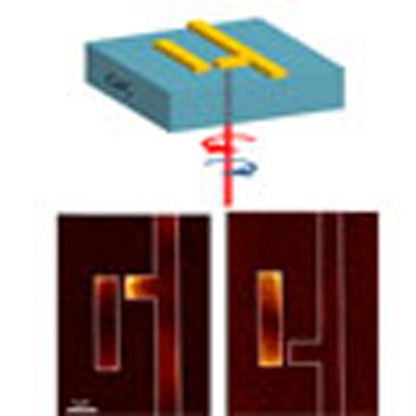

Just as left or right hands that lend the name to the fascinating geometric concept of chirality, certain types of molecules cannot be brought into congruence with their mirror image enantiomer. From quartz crystals to organic sugars and nucleic acids, such ‘chiral' molecules are ubiquitous in the physical world. For reasons not yet entirely understood, biological molecules tend to occur, predominantly, in either left- or right-handed chiral forms^1^. Similarly, due to their vector nature, light waves also naturally possesses two forms of handedness and can exist as left and right circularly polarized (LCP and RCP, respectively) waves. So it is not surprising that one of the earliest discovered manifestations of chirality belongs to optics: chiral materials are responsible for the phenomenon of optical activity that results in the rotation of the polarization state of light. Polarization rotation occurs because light of opposite handedness perceives different refractive indices and absorption coefficients when propagating through any homochiral material. These effects manifest themselves as unequal absorption rates (circular dichroism) and unequal phase advances (circular birefringence) for LCP and RCP light.

Because chiral response in naturally occurring materials is quite weak, with circular birefringence of the order of fine structure constant *α*≅1/137 (refs 2, 3), alternative approaches based on metamaterials and metasurfaces have been recently explored^4^. The most straightforward way of producing strong circular dichroism and birefringence is to mimic natural helical molecules, such as glucose, by synthesizing fully three-dimensional (3D) chiral metamolecules that look like helices^5^,^6^,^7^,^8^ Such artificial structures exhibit strong transmission difference between LCP and RCP polarization states of light, as well as handedness-dependent non-linear response^9^. However, an alternative approach to achieving circular dichroism that does not rely on 3D nature of the metamolecules has recently emerged. Fully two-dimensional (2D) metamaterials, also known as metasurfaces^10^,^11^,^12^,^13^,^14^,^15^,^16^,^17^,^18^, comprised of planar-chiral plasmonic metamolecules that are just nanometres thick, have been shown to exhibit chiral dichroism in transmission (CDT). Theoretical calculations^14^,^19^,^20^ indicate that this surprising effect relies on finite non-radiative (Ohmic) losses of the metasurface. In the absence of such losses on the nanoscale, the CDT defined as the difference Δ*T*_CD_=*T*_R_−*T*_L_ between the transmission coefficients *T*_R_ and *T*_L_ of the RCP and LCP waves must identically vanish. This surprising theoretical prediction has never been experimentally verified because of the challenge of measuring non-radiative loss on the nanoscale.

In this study we use a combination of nanoscale-characterization techniques to demonstrate that the RCP and LCP states of the incident light produce drastically different distributions of optical energy and Ohmic heat dissipation in the 2D chiral nanoantennas, thereby producing a strong chiral dichroism in absorption (CDA) Δ*A*_CD_=*A*_R_−*A*_L_. A planar-chiral metasurface, along with its chiral enantiomer, was designed to maximize the CDA in mid-infrared wavelength range^21^. The CDA gives rise to the CDT observed experimentally in the far-field measurements. We then use scattering-type near-field-scanning optical microscopy (s-SNOM)^22^,^23^ to map the optical energy distribution on the nanoantennas and their enantiomers in response to the RCP and LCP light. Photo-expansion microscopy^24^,^25^,^26^,^27^,^28^, also known as AFM-IR, was then utilized to experimentally demonstrate drastically different Ohmic heating of the nanoantennas under RCP and LCP light illumination.

## Results

### Design and theoretical analysis of the metasurface

**Figure 1:**
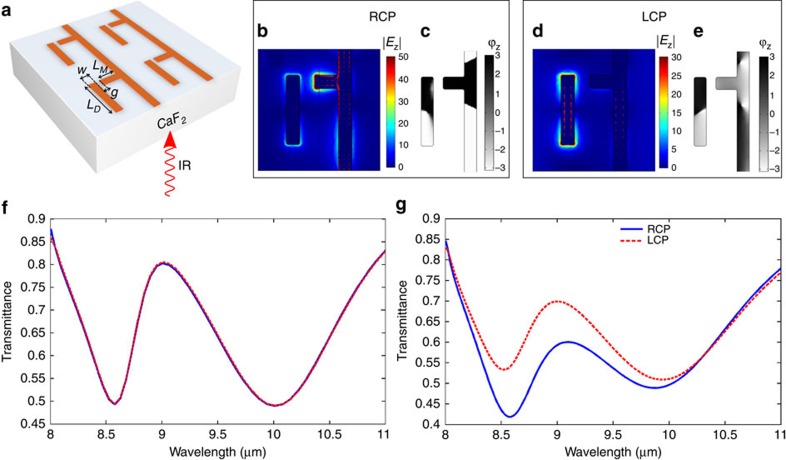
Circular dichroism in a planar-chiral plasmonic metasurface caused by Ohmic losses. (**a**) Schematic of a periodic metasurface (four unit cells shown) illuminated by circularly polarized light from under the CaF_2_ substrate. (**b**–**e**) Surface current (arrows) and colour-coded amplitude (**b**,**d**) and grey-scale phase (**c**,**e**) of the normal electric field *E*_z_ from COMSOL simulations of normally incident RCP (**b**,**c**) and LCP (**d**,**e**) light waves. (**f**,**g**) Transmission spectra of the metasurface for the cases of RCP (blue solid line) and LCP (red dashed line) excitations without (**f**) and with (**g**) Ohmic loss in the metal. Simulation parameters: *P*_x_=*P*_y_=6 μm, *L*_M_=1 μm, *L*_D_=3.3 μm, *w*=0.6 μm, *g*=0.4 μm. Frequency-dependent refractive index of gold 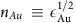 was obtained from ref. 44, and  frequency-independent refractive index 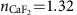 of the substrate was used.

The specific plasmonic metasurface shown in Fig. 1a consists of a square array of asymmetric unit cells. Each unit cell consists of two resonant gold elements, (i) a vertical dipole nanoantenna and (ii) a horizontal monopole nanoantenna^29^,^30^, connected to a vertical plasmonic wire running across the entire metasurface. In the absence of the near-field interaction between the nanoantennas, their respective plasmonic resonances appear at the wavelengths *λ*_D_≡2*πc*/*ω*_*y*_ and *λ*_M_≡2*πc*/*ω*_x_, respectively. The near-field interaction between the two antennas reduces the metasurface's symmetry, making it planar-chiral because of the absence of mirror-reflection symmetry^14^,^31^,^32^. The minimal mathematical description of the nanoantennas' interaction with each other and with the incident light wave with complex-valued electric field amplitudes (*E*_*x*_, *E*_*y*_) is given by the following set of equations obtained from the temporal coupled mode theory^32^,^33^,^34^:


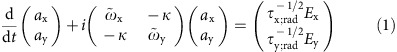


where, *a*_*x,y*_ are the electric dipole moments' amplitudes, and 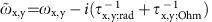 are the complex-valued frequencies of the respective plasmonic resonances that take into account their radiative (*τ*_x,y;rad_) and Ohmic (*τ*_x,y;Ohm_) lifetimes. The near-field coupling coefficient κ between the two resonances accounts for the planar-chiral nature of the metasurface, giving rise to the proportional CDT (see the ‘Methods' section for details):


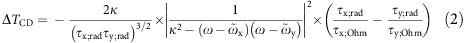


Several observations can be made from equation (2). (a) No CDT is possible without Ohmic loss in at least one of the two plasmonic modes, and (b) the CDT is maximized if the two modes have very different ratios between their Ohmic and radiative lifetimes (*τ*_x;rad_/*τ*_x;Ohm_≠*τ*_y;rad_/*τ*_y;Ohm_) and nearly equal resonant frequencies (*ω*_x_≈*ω*_y_). The latter condition informs our specific choice of the metasurface because the monopole nanoantenna's mode is known^21^ to have a much longer radiative lifetime than the dipole antenna's mode, and the two can be spectrally overlapped.

To confirm these simple analytic predictions, we have carried out first-principles COMSOL simulations of the metasurface shown in Fig. 1. By artificially removing Ohmic losses from the simulation, we observed from Fig. 1f that indeed *T*_R_=*T*_L_ is in accordance with equation (1) and in agreement with earlier theoretical predictions^13^,^19^. Therefore, a non-dissipative planar metasurface cannot exhibit optical activity despite the fact that the near-field optical intensities produced by the incident LCP and RCP light waves are markedly different in their spatial distribution and intensity, as can be observed from Fig. 1b–e. Ohmic loss drastically changes this situation and induces considerable CDT as shown in Fig. 1g. The qualitative physical explanation of the CDT is that different optical intensity and electric current distributions induced by the RCP and LCP light, that is, weak excitation of the vertical nanoantenna for the LCP light and much stronger excitation of the horizontal nanoantenna by the RCP light as shown in Fig. 1b,d, leads to strong CDA (Δ*A*_CD_>0) in the spectral proximity of the plasmonic resonance of the nanoantennas. Therefore, Δ*T*_CD_<0 is expected because the transmission of the RCP light is weakened by the light absorption in the metal (or, as it would be relevant for microwave metasurfaces^13^, in the substrate). This explanation directly follows from the analytic model given by equation (1), as detailed in the ‘Methods' section. From symmetry considerations we also conclude that the signs of Δ*A*_CD_ and Δ*T*_CD_ are reversed for the metasurface enantiomer obtained by the *y*→−*y* symmetry transformation, which formally reverses the sign of κ in equation (1). Note that the effect of the asymmetry between the substrate (CaF_2_, with the refractive index 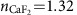) and the air superstrate is extremely weak as observed from Fig. 1f.

### Fabrication and optical characterization

Despite its apparent simplicity and qualitative appeal, this conceptual sequence—from different near-field distributions produced by RCP/LCP light to differential Ohmic dissipation to CDT—has never been experimentally demonstrated because of the challenge of measuring the near-field distribution and Ohmic loss on the nanoscale. Here, we provide the experimental evidence on the dissipation's role in inducing chiral dichroism by using two complementary AFM-based techniques for directly comparing the effects of the two circular light polarizations on the two metasurface enantiomers: (i) transmission-mode s-SNOM for measuring the distribution of optical fields on the nanoantennas^35^,^36^,^37^, and (ii) AFM-IR^24^,^25^,^26^,^27^,^28^ for measuring Ohmic heating of the metal.

**Figure 2:**
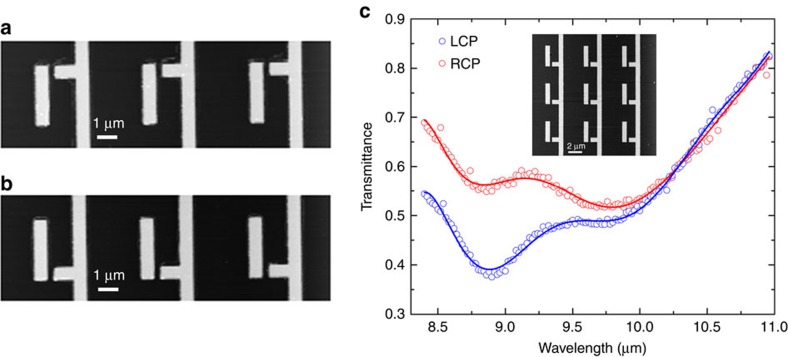
Far-field measurements of CDT in a planar-chiral metasurface. (**a**,**b**) AFM image of the enantiomers fabricated on top of CaF_2_ substrate. The two enantiomers (top and bottom) are mirror images of each other. (**c**) Experimental far-field transmission for the structure shown in the inset (and also corresponding to (**b**)) revealing the CDT caused by the Ohmic loss. Open circles represent the experimental points for LCP (blue) and RCP (red) excitations; solid lines are for guiding the eye. Inset shows SEM image of the enantiomer studied.

The nanoscale topographies of the two metasurface enantiomers (referred from here on as upper and lower) are shown in Fig. 2a,b. They are fabricated on top of a mid-infrared transparent CaF_2_ substrate using e-beam lithography, followed by gold deposition and the lift-off. Circularly polarized light pulses from a tunable quantum cascade laser (QCL) were used to illuminate the lower enantiomer (see the ‘Methods' section for fabrication and measurements' details). The transmission spectra *T*_R_(*λ*) and *T*_L_(*λ*) shown in Fig. 2c were measured across the 8.25 μm<*λ*<11 μm spectral range. The experimental results clearly demonstrate strong CDT, which is most pronounced near to the horizontal nanoantenna's resonance *λ*_M_≅9 μm. Specifically, the much lower transmission of the LCP light correlates with higher Ohmic losses of the LCP for the lower enantiomer. Note that the opposite is true for the upper enantiomer, see Fig. 1g. While a substrate can, in principle, induce small CDT, our numerical simulations shown in Fig. 1f indicate that the effect is too small to be measurable.

**Figure 3:**
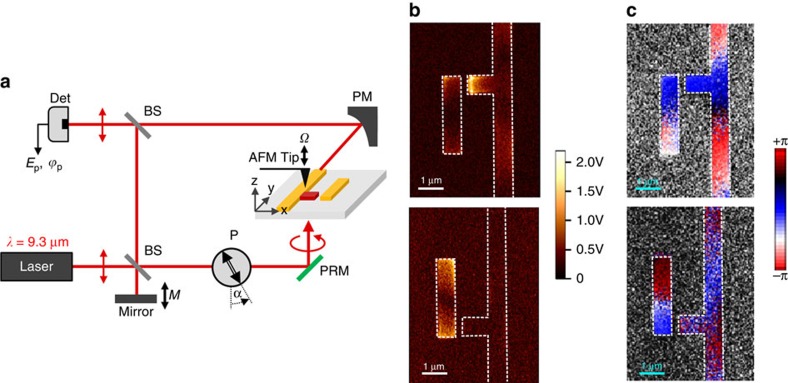
s-SNOM measurements of the optical field above two planar-chiral metasurfaces illuminated by RCP light. (**a**) Schematics of the experimental setup. (**b**) Measured amplitude 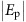 and (**c**) phase of the normal electric field *E*_p_ at *λ*=9.3 μm. White dashed lines in **b**,**c** outline the geometry of the structure. BS, beam splitter; Det, pseudoeheterodyne detection; P, linear polarizer; PM, parabolic mirror; PRM, infrared phase retarding mirror.

For the field-scanning s-SNOM experiments, the structure was illuminated through the CaF_2_ substrate by RCP light generated by a CO_2_ laser at *λ*=9.3 μm (transmission-mode s-SNOM^35^,^36^,^37^). Schematics of the experimental setup are shown in Fig. 3a (see the ‘Methods' section for measurement details). The s-SNOM is based on an atomic force microscope (AFM) where a sharp dielectric tip locally scatters the local near fields on the sample surface. While the sample is scanned, the scattered light is detected interferometrically using a p-polarized reference beam, and amplitude and phase maps, *E*_p_ and *ϕ*_p_, of the near-field distribution of both enantiomers are obtained. In case of the presented structure, these maps mainly correspond to the vertical near-field component, *E*_z_ [Fig f4](see Fig. 5 in the ‘Methods' section section). The experimental results presented in Fig. 3b,c clearly reveal that the near-field optical intensity distribution strongly depends on the handedness of the enantiomer for a given handedness of incident light. In more detail, we observe the fundamental dipolar mode on the dipole antenna for both enantiomers, exhibiting strong amplitude signals at the rod ends and a phase jump at the rod center. The monopole antenna shows strong amplitude signal only for the upper enantiomer, while the fields completely vanish at the monopole antenna for the lower enantiomer. From the abovementioned symmetry considerations, it follows that the response of the lower enantiomer to the RCP light is identical to the response of the upper enantiomer to the LCP light. We, thus, have experimentally demonstrated for the first time that the near-field intensity distribution near a given planar-chiral metasurface strongly depends on light's handedness^32^,^38^.

The qualitative explanation of this effect rests on the observation that, depending on the enantiomer, the vertical dipole nanoantenna (which is primarily excited by the *E*_y_ component of the incident laser beam) either polarizes or de-polarizes the horizontal monopole nanoantenna excited by the *E*_x_ field component. This effect is most pronounced for CP light when the frequencies of the two modes are spectrally matched to each other and to the incident laser's frequency (*ω*≈*ω*_x_≈*ω*_y_), so that the phases of *a*_x,y_ with respect to *E*_x,y_ are approximately the same. Therefore, the ±*π*/2 phase shift between *E*_x_ and *E*_y_ (depending on light's handedness) results in either constructive or destructive interference depending on the enantiomer (that is, the sign of κ in equation (1)). This theoretical argument is validated by the excellent agreement between the experimental results presented in Fig. 3b,c, and the results of numerical modelling presented in Fig. 1b–e.

### Measurements of polarization-dependent Ohmic heating

We proceeded to demonstrate that the distinct optical intensity distributions produced by the two circular light polarizations shown in Fig. 3 give rise to correspondingly different Ohmic losses inside the plasmonic metasurface. This was accomplished by using the AFM-IR technique, which relies on detecting thermal expansion of polymers and other materials on nanoscale by observing cantilever deflection of the AFM. While AFM-IR technique has originally been developed for nanospectroscopy of organic compounds^24^,^25^,^28^, it can also be used to image local heat generation in nanoantennas by observing the expansion of the gold itself, or an infrared-transparent polymer film in contact with nanoantennas^26^,^27^. Polyethylene has virtually no infrared absorption at the laser wavelength, and its heating is produced entirely by Ohmic dissipation in nanoantennas.

**Figure 4 f4:**
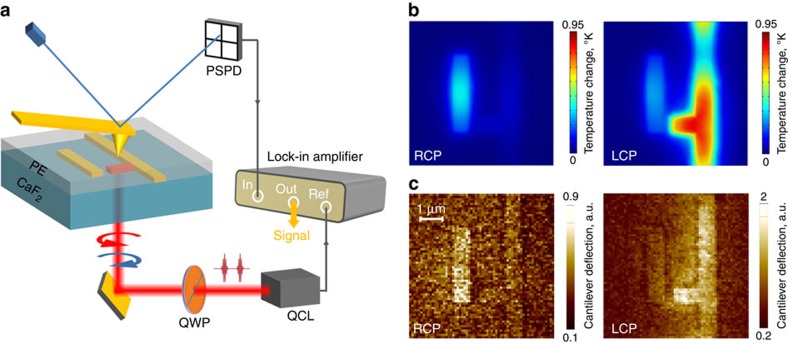
AFM-IR measurements of circularly dichroic thermal expansion of the metasurface. (**a**) Experimental setup. Light from a QCL passes through a quarter-wave plate (QWP) and illuminates the sample from CaF_2_ substrate. Local Ohmic heating of nanoantennas causes local heating and expansion of the polyethylene (PE) film and leads to cantilever deflection, which is detected by observing laser tracer beam deflection from cantilever, using position-sensitive photodetector (PSPD) and a lock-in amplifier, referenced by the QCL pulse repetition frequency. (**b**) COMSOL simulations of the temperature increase distribution in the polyethylene film at the end of a square QCL pulse with time duration *T*=200 ns and peak intensity *I*=1 kW cm^−2^ tuned to *λ*=9.1 μm, corresponding to the experimental conditions. (**c**) AFM-IR cantilever deflection on top of a PE-coated sample excited with RCP (left panel) and LCP (right panel) laser pulses at normal incidence through the CaF_2_ substrate.

For our measurements, we evaporated a 100-nm thick layer of polyethylene on top of the metasurface made of the lower enantiomer shown in Fig. 2b. The layer of polyethylene helps us to improve the photo-expansion signal because the linear thermal expansion coefficients of polyethylene are over an order of magnitude higher than that of gold. Circularly polarized 200-ns-long-light pulses at *λ*=9.1 μm from a tunable QCL were used to illuminate the sample through the CaF_2_ substrate at normal incidence with *I*≅1 kW cm^−2^ peak intensity on the sample (see Fig. 4a and the ‘Methods' section for the details of the experimental setup and measurements). Figure 4b shows the results of COMSOL simulations of the temperature distribution in the polyethylene film at the end of the LCP and RCP laser pulses. Because thermal diffusion length in polyethylene is well below 100 nm during the laser pulse polyethylene, heating is found to be highly localized and to closely follow the distribution of Ohmic dissipation (that is, the current density) in the metal nanoantennas shown in Fig. 1b–e.

Figure 4c shows the experimentally measured AFM cantilever deflection amplitude at different areas of the sample. The cantilever deflection is directly proportional to temperature increase in the sample during the laser pulse. The experimental data are in excellent agreement with theoretical predictions shown in Fig. 4b. It confirms that the magnitude and spatial distribution of the Ohmic heating of a chiral 2D metasurface markedly depends on the handedness of light. For example, the vertical dipole nanoantenna is extinguished for resonant LCP illumination. Considerably less polarization-induced asymmetry is induced by off-resonant illumination (see Supplementary Fig. 1 for thermal expansion images, and Supplementary Note 1 with Supplementary Fig. 2 for theoretical interpretation). These results represent the first direct experimental evidence of Ohmic loss origin of CDT in a planar-chiral plasmonic metasurface.

## Discussion

In conclusion, by using three distinct experimental techniques (two near-field and one far-field), we have demonstrated that optical activity in planar-chiral plasmonic metasurfaces is unambiguously correlated with circularly dichroic Ohmic loss, which is in turn caused by circularly dichroic near-field distribution. The direct mapping of nanoscale optical fields and absorption rates were carried out for two chiral metasurface enantiomers illuminated by circularly polarized infrared light. Although the importance of this work is primarily fundamental, it also paves the way for practical applications, such as the development of novel optical detectors for Stokes parameters' polarimetry, as well as for surface-enhanced enantiomeric sensing of chiral biological molecules^31^,^38^,^39^. The design principles behind achieving the large circularly dichroic effects in plasmonic metasurfaces that are established and experimentally verified in this study will be of great interest to a variety of researchers, especially in the field of biological sensing and novel infrared optical devices.

## Methods

### Fabrication of plasmonic planar-chiral metasurfaces

Planar-chiral plasmonic metasurfaces were fabricated on infrared-transparent CaF_2_ substrates using electron beam lithography, followed by a metal lift-off. Polymethyl methacrylate electron beam resist spun at 1,700 r.p.m. for 30 s was pre-baked for 1 min, followed by the deposition of a thin (∼3–5 nm) gold–paladium conductive layer using sputtering. The desired structures were written using a JEOL 6000 EBL system at 450 nC cm^−2^, and developed in 1:3 MIBK:IPA solution for 60 s. After development, the sample was washed with IPA and water, followed by a brief oxygen–plasma cleaning step (100 W for 5 s) to prepare for metal deposition. A 60-nm thick gold layer was thermally deposited following a 5-nm thick Cr adhesion layer. The sample was then immersed in acetone for lift-off, followed by oxygen–plasma cleaning.

### s-SNOM measurements of the field distribution at the metasurface

Our s-SNOM setup (see Fig. 3a for a schematic) is based on an atomic force microscope, where commercial silicon tips (NanoWord, Arrow-NCR-50) were used to locally scatter the near fields on the sample surface. The sample and tip were illuminated from below at normal incidence with a weakly focused, right-handed circularly polarized CO_2_ laser beam at a wavelength *λ*=9.3 μm (transmission-mode s-SNOM^35^,^36^,^37^), thus ensuring homogeneous illumination of a relatively large area on the sample without phase-retardation effects. Using a parabolic mirror, we collected the scattered light in the *x*-direction at an angle of 60° from the surface normal. The scattered light was then interfered with a phase-modulated, vertically (z-) polarized reference beam at the infrared detector and recorded simultaneously with the sample topography. Background contributions could be fully suppressed by vertical tip oscillation at a frequency *Ω*=250 kHz (tapping-mode AFM) and subsequent higher harmonic demodulation of the detector signal at 3*Ω* (ref. 40). In this case, the vertical polarization of the reference beam selected the p-component of the scattered light. Using a pseudoheterodyne detection module (www.neaspec.com) the amplitude 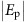 and phase *ϕ*_p_ of the scattered light were measured for each scanning point.

Recently, it was found that there are different scattering mechanism in s-SNOM imaging of metal antennas: (i) direct scattering of the antenna near fields by the tip into the far field (detector) and (ii) scattering of the antenna near fields by the tip via the antenna itself^41^,^42^. When imaging resonant linear antennas, excitation with s-polarization and detecting the s-component of the scattered light is mainly based on the mechanism (ii), while detecting the p-component is mainly based on the mechanism (i), where the latter was found to essentially yield the z-component, *E*_z_, of the near fields on the antenna surface^42^. In case of the presented metasurface, the p-component of the scattered field mainly yields the vertical (z) component, *E*_z_, of the near fields, as it was found by comparing the experimental images with numerical calculations^37^. The vertical (z) component, *E*_z_, is closely related to the magnitude and phase of the surface-charge density in metal antennas, so that the near-field maps shown in Fig. 3 allow for unambiguous identification of the antenna modes.

### Comparison of numerical field profiles and s-SNOM measurements

**Figure 5:**
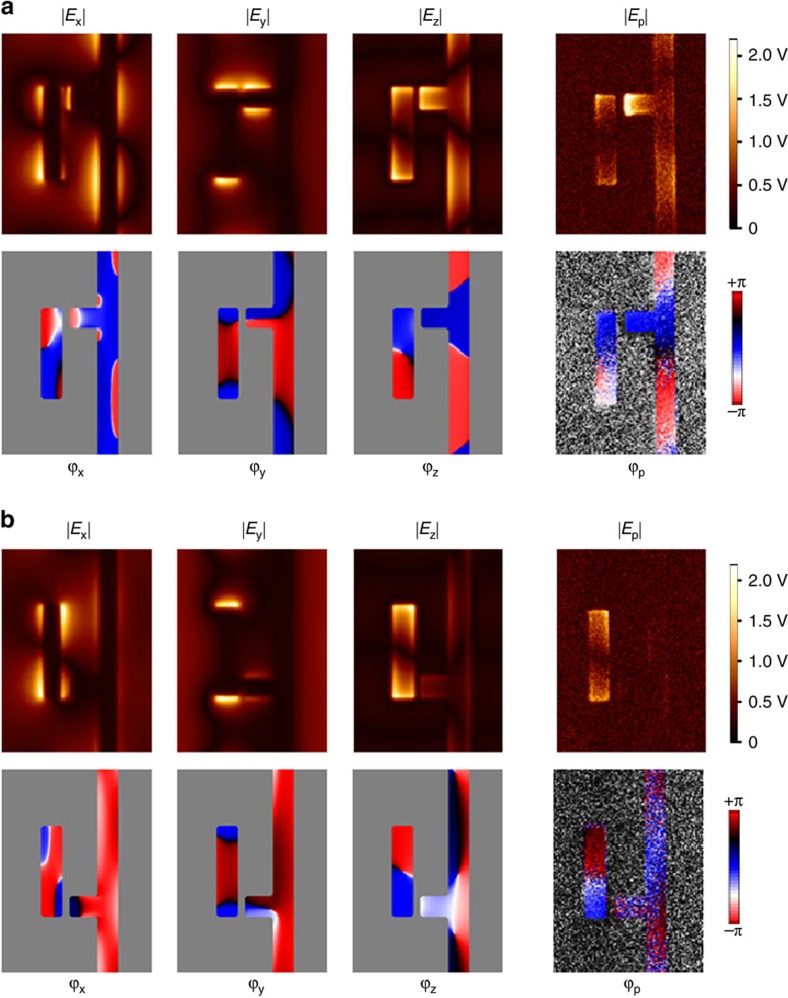
Comparison between numerical calculations. Displaying *E*_x_, *E*_y_, and *E*_z_ near-field components at a height of 50 nm above the structures (three left subplots) and the experimentally recorded near-field maps, showing the p-component of the scattered field, *E*_p_ (right subplots). The comparison shows that the experimental maps mainly yield the vertical near-field component, *E*_z_. (**a**,**b**) correspond to the two enantiomers of the metasurface. The colour scheme for the simulations results is the same as for the experimental results.

As the s-SNOM imaging relies on interferometric detection of the scattered light using a p-polarized reference beam, the correspondence between the experimental measured maps of the field amplitude, *E*_p_ and phase, *ϕ*_p_, and the numerically calculated fully vectorial field profiles should be established. As can be seen from Fig. 5, in case of the structure presented, comparison of the experimental and numerical maps clearly reveals that the measured field components correspond to the vertical *E*_z_ near-field component of the electric field.

### AFM measurements of thermal expansion

A pulsed mid-infrared QCL (Daylight Solutions, Inc.; tuning range of 900–1,200 cm^−1^) operating at 9.1-μm wavelength was used to illuminate the sample. The light from the QCL, originally linearly polarized, was converted to LCP or RCP light using an achromatic quarter-wave plate (Altechna Inc., 2-IRPW-ZO-L/4-8000-C). The laser was operated with 200-ns pulses with the peak power of 300 mW at a repetition frequency of ∼180 kHz, in resonances with the second bending mode of the AFM cantilever. To increase the photo-expansion signal, a 100-nm thick polyethylene film was thermally evaporated on top of the nanoantennas. The sample was positioned on the AFM stage with the polyethylene-coated metal nanoantennas being on top, right below the AFM tip. Laser radiation was incident normally onto the sample from below from the CaF_2_ substrate. The beam was focused to a 100-μm radius spot using ZnSe lens with the convergence half-angle of 4^o^. The QCL peak intensity on the sample surface was estimated to be ∼1 kW cm^−2^.

The AFM system was operated in contact mode. Gold-coated ContGB-G (Budget Sensor) AFM cantilevers were used for measurements. Thermal sample expansion during the laser pulse exerted a force on the AFM cantilever tip that leads to cantilever deflection. The cantilever deflection amplitude was measured by a position-sensitive photodetector and sent to a lock-in amplifier (Stanford Research, SR844) using QCL pulse repetition rate as a reference. The lock-in integration time was set at 10 ms. The output from the lock-in amplifier was used to form 64 × 64 pixel images shown in Fig. 3b by raster scanning the sample at the speed of 0.2 Hz. In our Multiphysics COMSOL simulations, which combined the simulation of the laser-induced metasurface heating and thermal conduction, the following thermal diffusivities were used for gold, CaF_2_ substrate and polyethylene: *D*_Au_=1.28 × 10^−4^ m^2^ s^−1^, *D*_sub_=3.6 × 10^−6^ m^2^ s^−1^, and *D*_PE_=10^−7^ m^2^ s^−1^, respectively.

We have also measured (not shown) the metal expansion directly, that is, without the PE polymer layer, at the resonant (*λ*_R_=9.1 μm) and at the non-resonant (*λ*_NR_=10.3 μm) wavelengths. Strong handedness-dependent expansion of the monopole antenna and the adjacent area of the continuous nanowire was found only at *λ*=*λ*_R_, whereas much weaker metal expansion was observed at *λ*=*λ*_NR_ for both circular light polarizations.

### Far-field measurements of circular dichroism in transmission

A pulsed mid-infrared QCL (Daylight Solutions, Inc.; tuning range of 900–1,200 cm^−1^) was used as a light source. Linearly polarized light from QCL passed through achromatic quarter-wave plate (Altechna Inc., 2-IRPW-ZO-L/4-8000-C) for polarization control and a numerical aperture 0.5 reflective objective lens (Edmund Optics, Adjustable ReflX Objective 36X/0.5NA IR FIN) to the sample. Transmitted light from the sample was collected and collimated by a 3-in focal length plano-convex ZnSe lens and refocused by another identical lens on a liquid nitrogen-cooled mercury–cadmium–telluride photodetector. The transmission spectral data from the metasurface were normalized using transmitted signal from a bare CaF_2_ substrate.

### Analytic coupled mode theory of circular dichroism in planar-chiral metasurfaces

The temporal coupled mode theory expressed by equation (1) is the minimal description of a metasurface in terms of its electromagnetic resonances that enables the calculation of transmission and reflection coefficients for arbitrarily polarized normally incident light wave with electric field amplitudes (*E*_x_, *E*_y_). For example, the reflectivity matrix 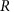 is obtained from the following equation:


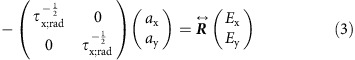


by continuity, the transmission matrix is given by 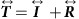, where, 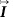 is a unity matrix. The state of polarization of the LCP/RCP light propagating in the z-direction is given by the following vectors:


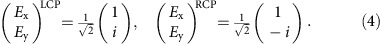


The CDT given by Δ*T*_CD_=*T*_R_−*T*_L_ can be expressed as 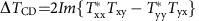, revealing that if the transmission matrix can be diagonalized by rotating its axis by an arbitrary angle *θ*, then Δ*T*_CD_=0. The frequency-dependent rotation angle *θ* is given by the following expression:


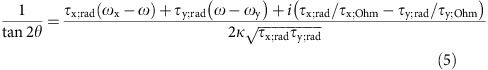


from which, it follows that a real-valued *θ* exists as long as 
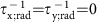
, that is, the Ohmic losses vanish^14^,^19^.

The vanishing of the CDT in the absence of Ohmic losses can be understood by expressing Δ*T*_CD_=*T*_LR_−*T*_RL_, where, *T*_LR_ and *T*_RL_ are the transmission conversion efficiencies from RCP to LCP, and from LCP to RCP, respectively^13^,^43^. The markedly different charge distributions on the two nanoantennas under RCP and LCP illuminations (see Fig. 1b–e for amplitude and phase information) are formally equivalent to 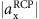 produced under resonant RCP illumination being much larger than 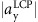 produced under either LCP illumination (note, from Fig. 1b that 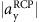 is negligible under RCP illumination). Nevertheless, the x-polarized monopole antenna resonance with the amplitude 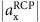 couples to LCP light with the same efficiency as the y-polarized dipole antenna resonance with the amplitude 
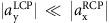
 couples to RCP light. The reason for which is the much lower radiative rate of the monopole antenna: 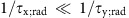. This qualitative argument explains why *T*_LR_=*T*_RL_ and, therefore, Δ*T*_CD_=0 despite strong differences in the near-field distributions is shown in Fig. 1.

For non-vanishing Ohmic losses, the total absorption coefficients for the LCP and RCP illuminations are


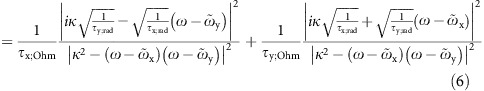



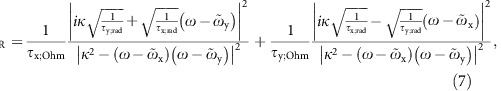


respectively, where the two terms in the sum represent the contributions of the monopole and dipole nanoantennas, respectively. The simplest limiting case corresponds to the matched plasmonic resonances of the two nanoantennas (*ω*_x_=*ω*_y_≡*ω*_R_) and the following hierarchy of radiative and non-radiative losses: 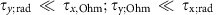. In this limiting case the expression for the absorption rates at resonance (*ω*=*ω*_R_) can be simplified by noting that most of the absorption happens at the monopole antenna:


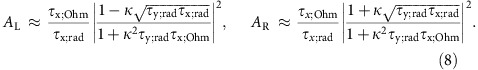


It clearly follows from these expressions that (a) larger loss (and, correspondingly, lower transmission) occurs for the RCP light in the case of the upper enantiomer with *κ*>0 shown in Fig. 1a; (b) the situation reverses for the lower enantiomer (see Fig. 2, bottom row) which is characterized by *κ*<0 as explained in the text.

### Data availability

The data that support the findings of this study are available from the corresponding author on request.

## Additional information

**How to cite this article:** Khanikaev, A. B. *et al*. Experimental demonstration of the microscopic origin of circular dichroism in two-dimensional metamaterials. *Nat. Commun.* 7:12045 doi: 10.1038/ncomms12045 (2016).

## Supplementary Material

Supplementary Information Supplementary Figures 1 & 2 and Supplementary Note 1.
