# Supplementary material for: Experimental demonstration of the microscopic origin of circular dichroism in two-dimensional metamaterials
Source: Nat Commun. 2016 Jun 22;7:12045. doi: 10.1038/ncomms12045 (PMC4917967; doi:10.1038/ncomms12045)
Supplement: Supplementary Information — Supplementary Figures 1 & 2 and Supplementary Note 1. [file ncomms12045-s1.pdf]

Supplementary Figures

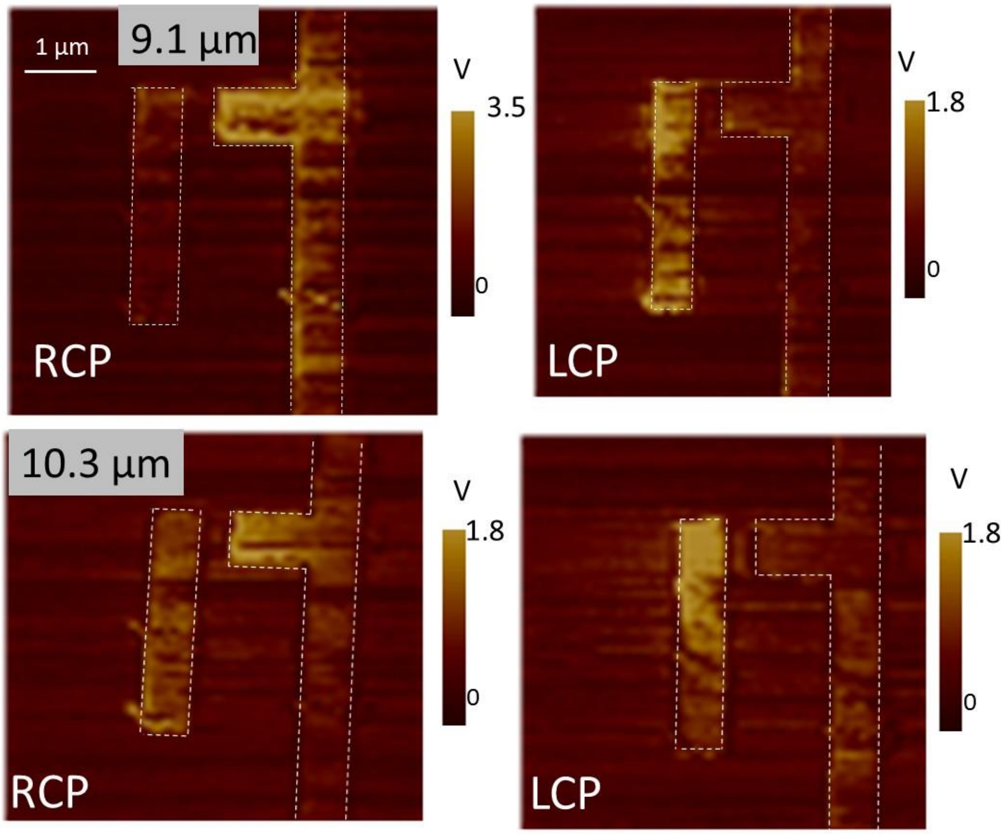

Supplementary Figure 1. Top row: Resonant excitation at $\lambda_R = 9.1\mu m$. This figure is similar to Figs.4b,c, except that metal expansion was measured directly, i.e. without adding a transparent expanding polymer on top of the metasurface. The RCP light extinguishes the vertical (dipole) antenna while strongly heating the monopole antenna. The LCP light is heating the dipole antenna only. Bottom row: off-resonant excitation at $\lambda_{NR} = 10.3\mu m$. We observe that (a) the heating is much smaller than in the resonant case, and (b) the dipole antenna is excited by both RCP and LCP light.

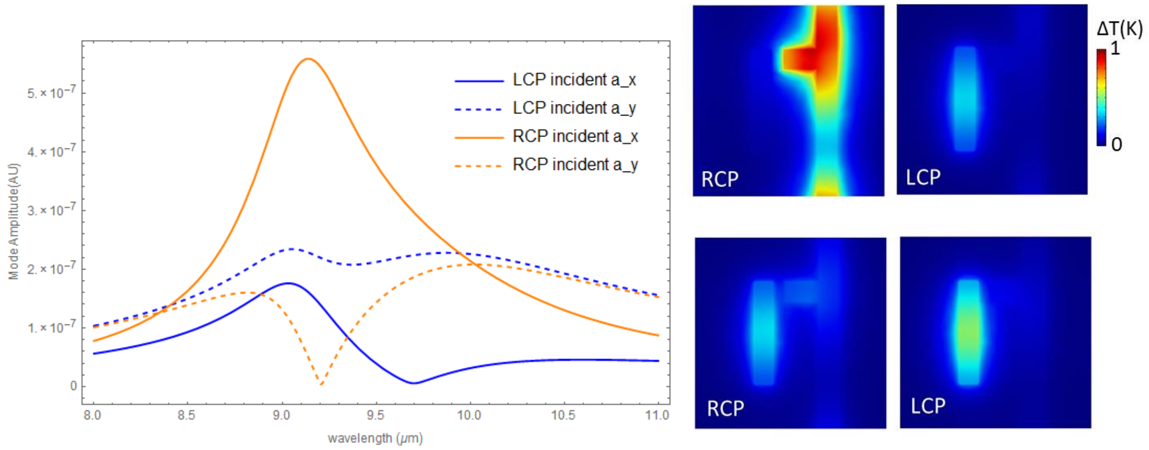

Supplementary Figure 2. Left panel: amplitudes of the monopole antenna excitation $a_x(\lambda)$and the dipole antenna excitation $a_y(\lambda)$ plotted as the function of the excitation wavelength λ for RCP and LCP illumination. The analytic model is described in the manuscript. Note that a_x is larger under the RCP illumination, both on and off resonance. Right panel: COMSOL modeling of metal heating at $\lambda_R = 9.1\mu m$ (top row) and at $\lambda_{NR} = 10.3\mu m$ (bottom row) by the two circular polarizations. COMSOL results illustrate the predictions of the analytic model: the monopole antenna is always stronger excited by RCP. Parameters used in the analytic model (see the manuscript for parameter definitions): monopole and dipole antenna resonance frequencies and radiative lifetimes: $\omega_{\mathrm{x}} = 1.086 \times 10^3 \mathrm{cm}^{-1}$, $\tau_{\mathrm{x,rad}} = 285\mathrm{fs}$, $\omega_{\mathrm{y}} = 1.031 \times 10^3 \mathrm{cm}^{-1}$, $\tau_{\mathrm{y,rad}} = 50\mathrm{fs}$. The Ohmic lifetimes of the two resonances are assumed to be $\tau_{\mathrm{x},Ohm} = \tau_{\mathrm{y},Ohm} = 210\mathrm{fs}$. The coupling coefficient between the two resonances is assumed to be $\kappa = 43\mathrm{cm}^{-1}$.

Supplementary Note 1: Metasurface heating using off-resonance laser excitation

Although it is not possible to control losses in the experiment, we have carried out additional experiments aimed at quantifying chirality-dependent Ohmic heating at the tuned laser wavelength of $\lambda_{NR} = 10.3\mu m$ (on the red side of the Fano resonance). To simplify measurements, we have imaged metal expansion directly, without coating the samples with the polyethylene film. Thermal expansion of bare metal due to Ohmic losses may also be detected by the AFM-IR technique, although the cantilever deflection signal in this case is smaller than in the case of the samples coated with the polymer film that are described in the main manuscript. Another challenge is that the variation of the surface morphology in the absence of the top polymer translates into a drift of the resonance cantilever frequency. The results of this AMF-IR imaging of the plasmonic metasurface not coated with PE and illuminated by RCP and LCP light at $\lambda_R = 9.1\mu m$ and $\lambda_{NR} = 10.3\mu m$ are shown in Supplementary Figure 1. Note that the enantiomer used for the measurements in Supplementary Figure 1 is shown in Fig.2(a) of the manuscript; it is different from the one that was optically characterized in Fig.2c.

The interpretation of the images shown in Supplementary Figure 1 is as follows. At the resonant wavelength $\lambda_R = 9.1\mu m$ (top row) the RCP light almost completely extinguishes the vertical (dipole) antenna (i.e. it appears completely dark in the AFM image of the metal expansion), whereas the LCP light does not. At the same time, the RCP light strongly excites the horizontal (monopole) antenna and the adjacent region of the vertical nanowire to which it is attached: both are very bright in the AFM image. The strong heating of the monopole antenna by the RCP light (note the range difference for the RCP and LCP cases) causes a drop in transmission shown in Fig.2c of the manuscript.

The situation changes dramatically for the off-resonance wavelength $\lambda_{NR} = 10.3\mu m$ (bottom row). Specifically, we find that (a) the Ohmic heating is strongly reduced compared with the resonant case (note the color bar range difference between the top and bottom rows), and (b) the dipole antenna is strongly excited by both circular polarizations of the incident light, i.e. its image is bright for both RCP and LCP light. In other words, the excitation of the dipole antenna by the RCP light is not suppressed at λ_{NR}.

It is worth noting that some visual asymmetry between RCP and LCP cases persists even at λ_{NR}. Specifically, the RCP light excites the monopole antenna while the LCP light does not. This property of planar chiral metasurfaces is confirmed by both analytic modeling (left panel of the Supplementary Figure 2) and by the COMSOL simulations of metal heating (right panel of the Supplementary Figure 2).
